# C1-Inhibitor protects from focal brain trauma in a cortical cryolesion mice model by reducing thrombo-inflammation

**DOI:** 10.3389/fncel.2014.00269

**Published:** 2014-09-09

**Authors:** Christiane Albert-Weissenberger, Stine Mencl, Michael K. Schuhmann, Irmak Salur, Eva Göb, Friederike Langhauser, Sarah Hopp, Nelli Hennig, Sven G. Meuth, Marc W. Nolte, Anna-Leena Sirén, Christoph Kleinschnitz

**Affiliations:** ^1^Department of Neurology, University Hospital WürzburgWürzburg, Germany; ^2^Department of Neurosurgery, University Hospital WürzburgWürzburg, Germany; ^3^Department of Neurology, University of MünsterMünster, Germany; ^4^Institute of Physiology I - Neuropathophysiology, University of MünsterMünster, Germany; ^5^CSL Behring GmbHMarburg, Germany

**Keywords:** traumatic brain injury, edema, blood-brain barrier, C1-inhibitor, contact-kinin system, inflammation, thrombosis

## Abstract

Traumatic brain injury (TBI) induces a strong inflammatory response which includes blood-brain barrier damage, edema formation and infiltration of different immune cell subsets. More recently, microvascular thrombosis has been identified as another pathophysiological feature of TBI. The contact-kinin system represents an interface between inflammatory and thrombotic circuits and is activated in different neurological diseases. C1-Inhibitor counteracts activation of the contact-kinin system at multiple levels. We investigated the therapeutic potential of C1-Inhibitor in a model of TBI. Male and female C57BL/6 mice were subjected to cortical cryolesion and treated with C1-Inhibitor after 1 h. Lesion volumes were assessed between day 1 and day 5 and blood-brain barrier damage, thrombus formation as well as the local inflammatory response were determined post TBI. Treatment of male mice with 15.0 IU C1-Inhibitor, but not 7.5 IU, 1 h after cryolesion reduced lesion volumes by ~75% on day 1. This protective effect was preserved in female mice and at later stages of trauma. Mechanistically, C1-Inhibitor stabilized the blood-brain barrier and decreased the invasion of immune cells into the brain parenchyma. Moreover, C1-Inhibitor had strong antithrombotic effects. C1-Inhibitor represents a multifaceted anti-inflammatory and antithrombotic compound that prevents traumatic neurodegeneration in clinically meaningful settings.

## Introduction

Traumatic brain injury (TBI) accounts for more than 10 million fatalities worldwide and is a leading cause of permanent disability (Hyder et al., 2007; Roozenbeek et al., 2013). Albeit TBI is of utmost socioeconomic relevance, its underlying pathophysiology is still incompletely understood and specific therapies are lacking (Roozenbeek et al., 2013). After the initial impact, which irretrievably destructs the adjacent brain regions, a self-propagating deleterious cascade is unleashed that causes secondary tissue damage (Shlosberg et al., 2010). Inflammation is one of the most relevant contributors to this cascade (Cederberg and Siesjo, 2010). Early after trauma the brain endothelium upregulates cellular adhesion molecules and this activation step enables trafficking of inflammatory cells (neutrophils, macrophages) from the blood stream to the sites of tissue damage (Schwarzmaier et al., 2013). Those peripheral cells together with resident cell populations (endothelial cells, microglia, astrocytes) produce myriads of highly active mediators such as cytokines and chemokines that perpetuate the inflammatory response (Schmidt et al., 2005). Another characteristic of severe brain trauma is structural disintegration of the blood-brain barrier, which in consequence leads to the formation of brain edema (Shlosberg et al., 2010). Excessive edema can damage otherwise healthy brain regions by compression and is a frequent cause of delayed neurologic deterioration in trauma patients. Pharmaceuticals able to substantially influence inflammation or edema formation in TBI are not available and decompressive surgery, which is a highly invasive procedure, failed to prove efficacy in trauma patients in a recent phase III trial (Cooper et al., 2011).

Apart from inflammation, microvascular dysfunction and progressive thrombus formation are increasingly recognized as important players in the pathophysiology of brain trauma and may account for the frequently observed immediate decline in regional cerebral blood flow which can also affect remote brain areas (Dietrich et al., 1996; Schwarzmaier et al., 2010; Prodan et al., 2013; Sillesen et al., 2013). Most interestingly, there is accumulating evidence of a tightly regulated interplay between thrombotic and inflammatory mechanisms during TBI (Schwarzmaier et al., 2010, 2013) and related CNS disorders such as ischemic stroke (Langhauser et al., 2012; Kleinschnitz et al., 2013), and this “thrombo-inflammation” might be accessible to specific therapeutic interventions (Nieswandt et al., 2011; Magnus et al., 2012).

The serine proteases coagulation factor XII (FXII) and plasma kallikrein together with their substrate kininogen build the contact-kinin system (Renné, 2012). The contact-kinin system fosters vascular permeability and inflammation by the formation of short-lived kinins while at the same time is linked to thrombus formation via the FXII-driven intrinsic coagulation cascade. All components of the contact-kinin system have been identified in the brain (Camargo et al., 1973; Kariya et al., 1985; Kizuki et al., 1994) and activation of the contact-kinin system has been described after TBI both experimentally as well as in humans (Auer and Ott, 1979; Trabold et al., 2010; Albert-Weissenberger et al., 2013). Hence, the different members of the contact-kinin system represent attractive targets to combat injury-induced inflammation and thrombosis.

C1-Inhibitor (C1-Inh) belongs to the superfamily of serine protease inhibitors called serpins (Davis et al., 2008). It acts as an important endogenous regulator of the contact-kinin system by blocking of activated FXII (FXIIa) and plasma kallikrein (Davis et al., 2010). Moreover, C1-Inh can directly interfere with the attraction of circulating leukocytes (Cai and Davis, 2003) and inhibits components of the complement system (Duehrkop and Rieben, 2014). Application of C1-Inh has proven to be beneficial in a variety of disorders associated with inflammation (Begieneman et al., 2012; Heydenreich et al., 2012; Mejia and Davis, 2012). In a previous publication, the group of De Simoni evaluated the effects of Cl-Inh following controlled cortical impact (CCI) brain injury in mice (Longhi et al., 2008, 2009). They showed that post-traumatic administration of the C1-Inh improved cognitive outcome and reduced histological damage after CCI, a model of focal and diffuse brain damage (Longhi et al., 2008, 2009). Importantly, they showed that C1-Inh treatment results in a better functional outcome.

To specifically answer the question whether C1-Inh, reduces blood-brain barrier breakdown, brain edema and lesion size in a focal TBI model, we used a cryolesion model that produces a standardized focal cortical lesion, breakdown of the blood-brain barrier and vasogenic brain edema (Raslan et al., 2012), key pathomechanisms associated with fatal outcome after focal clinical TBI. We show that plasma-derived C1-Inh protects from TBI in mice in a clinically relevant scenario by a combined anti-inflammatory and antithrombotic mode of action.

## Materials and methods

### Cortical cryolesion model

A total of 186 C57BL/6 mice (166 males, 22 females) were used in this study. All experiments were approved by institutional (University of Würzburg, Germany) and regulatory (local government of Lower Franconia, Bavaria, Germany) authorities. Cortical cryolesion was induced as described (Raslan et al., 2012). Briefly, mice were anesthetized with intraperitoneal injections of ketamine (0.1 mg/g) and xylazine (0.005 mg/g). Surgery was performed on the right parietal cortex after exposing the skull through a scalp incision. A copper cylinder with a tip diameter of 2.5 mm was filled with liquid nitrogen (−196°C) and placed stereotactically on the right parietal cortex (coordinates from bregma: 1.5 mm posterior, 1.5 mm lateral) for 90 s. Sham-operated animals went through the same procedure without cooling the copper cylinder. Animals were randomly assigned to the treatment groups by an independent person not involved in data acquisition. We analyzed all read-out parameters while being masked to the experimental groups.

### C1-inhibitor treatment

One hour after the induction of cortical cryolesion, mice received a single intravenous injection of human plasma-derived C1-Inh (Berinert®; CSL Behring GmbH) at a dose of 7.5 IU or 15.0 IU (Heydenreich et al., 2012). Control animals received equal volumes of isotonic saline (vehicle).

### Determination of lesion size

Twenty-four hours or 5 days after cryolesion, mice were sacrificed and mouse brains were quickly removed and cut in five 1 mm thick coronal sections using a mouse brain slice matrix (Harvard Apparatus). The slices were stained for 20 min at 37°C with 2% 2,3,5-triphenyltetrazolium chloride (TTC) (Sigma-Aldrich) in 1x phosphate buffered saline (PBS) to visualize the lesion. The lesion volume was calculated from the TTC stained slices using the ImageJ software (ImageJ software, National Institutes of Health, USA) (Raslan et al., 2010).

### Determination of brain edema and blood-brain barrier leakage

Brain edema formation was calculated using the wet weight-dry weight method (Langhauser et al., 2012). Briefly, brains were removed 24 h after cryolesion and a 6-mm-thick coronal section was dissected that included the traumatic area. The section was divided into an ipsilesional (injured) and contralesional (noninjured) part. The freshly collected tissue samples were weighted to assess the wet weight. After that, samples were dried for 72 h at 60°C and the dry weight was determined. The water content (expressed as percentage) in the ipsilesional and contralesional part was calculated using the following formula: ((wet weight—dry weight) / wet weight) × 100.

To determine blood-brain barrier leakage 100 μl of 2% Evans Blue tracer (Sigma Aldrich) diluted in 0.9% NaCl was i. v. injected 23 h after the induction of cryolesion (Langhauser et al., 2012). After 24 h mice were sacrificed and brains were quickly removed. A 6-mm-thick coronal section including the traumatic area was cut using a mouse brain slice matrix (Harvard Apparatus). The section was separated into an ipsilesional and contralesional part. Then, 300 μl formamide was added and incubated for 24 h at 55°C in the dark to extract the Evans blue dye. Tubes were centrifuged for 20 min at 16.000 g and 50 μl of the supernatant were transferred to a 96 well plate. Fluorescence intensity was determined in duplicates by a microplate fluorescence reader (Fluoroskan Ascent, Thermo Scientific) with an excitation at 610 nm and emission at 680 nm. The concentration for each sample was calculated from a standard curve.

### Real-time PCR studies

RNA was isolated from the whole ipsilesional hemisphere 24 h after trauma. Tissue homogenization, RNA isolation, and real-time PCR were performed as described (Kleinschnitz et al., 2010; Albert-Weissenberger et al., 2012). Briefly, total RNA was prepared with a Miccra D-8 power homogenizer (ART Prozess-& Labortechnik) using the TRIzol reagent (Invitrogen) and was quantified spectrophotometrically. Then, 250 μg of total RNA was reversely transcribed with the TaqMan Reverse Transcription Reagents (Applied Biosystems) according to the manufacturer’s protocol using random hexamers. Relative mRNA levels were quantified with the fluorescent TaqMan technology. PCR primers and probes specific for murine interleukin (IL)-1β (assay ID: Mm004344228_m1), tumor necrosis factor (TNF)α (assay ID: Mm00443258_m1), chemokine ligand 2 (CCL2) (assay ID: Mm00441242_m1), chemokine ligand 3 (CCL3) (assay ID: Mm00441259_g1), occludin (assay ID: Mm00500912_m1) and claudin-5 (assay ID: Mm00727012_s1) were obtained as TaqMan Gene Expression Arrays (Applied Biosystems). Glyceraldehyde 3-phosphate dehydrogenase (GAPDH) and β-Actin (TaqMan Predeveloped Assay Reagents for gene expression, part number: 4352339E and 4352341E; Applied Biosystems) were used as endogenous controls to normalize the amount of sample RNA. The PCR was performed with equal amounts of cDNA in the GeneAmp 7700 sequence detection system (Applied Biosystems) using the TaqMan Universal PCR Master Mix (Applied Biosystems). Reactions were incubated at 50°C for 2 min, at 95°C for 10 min followed by 40 cycles of 15 s at 95°C and 1 min at 60°C. Water controls were included to ensure specificity. Each sample was measured in triplicate and data points were examined for integrity by analysis of the amplification plot. The ΔΔCt method was used for relative quantification of gene expression as described (Livak and Schmittgen, 2001; Langhauser et al., 2012).

### Histology and immunohistochemistry

Immunohistochemistry was performed as previously described (Langhauser et al., 2012). Cryo-embedded brains were cut into 10-μm-thick slices using a cryostat (Leica). For staining of microglia/macrophages the slices were fixed in 4% PFA in PBS. Blocking of epitopes was achieved by pre-treatment with 5% bovine serum albumin (BSA) in PBS for 45 min to prevent unspecific binding. For the detection of activated microglia/macrophages the antibody (rat, diluted 1:100, Serotec MCA711, anti-CD11b) was diluted in PBS containing 1% BSA and incubated overnight at 4°C. Afterwards, slides were incubated with a biotinylated anti-rat IgG (BA-4001, Vector Laboratories) diluted 1:100 in PBS containing 1% BSA for 45 min at room temperature. Following treatment with Avidin/Biotin blocking solution (Avidin/Biotin Blocking Kit, Sp-2001, Vector Laboratories) to inhibit endogenous peroxidase activity, the secondary antibody was linked via streptavidin to a biotinylated peroxidase (POD) according to the manufacturer’s instructions (Vectorstain ABC Kit, Peroxidase Standard PK-4000, Vector Laboratories). Antigens were visualized via POD using the chromogen 3, 3′-Diaminobenzidin (DAB) (Kem-En-Tec Diagnostics), the slices were embedded in AquaTex (Merck) and digital images were acquired using a Nikon microscope Eclipse 50i equipped with the DS-U3 DS camera control unit and the NIS-Elements software (Nikon, Japan). In order to determine the number of macrophages and activated microglia, CD11b-positive cells were counted for each animal on the side of injury and on the contralateral side on five brain slices at 20x magnification. The numbers of CD11b-positive cells are expressed as cells/mm^2^. Negative controls for all immunohistochemical experiments included omission of either the primary or secondary antibody and gave no signals (not shown).

For the assessment of the thrombosis index, hematoxylin and eosin (H&E) staining on cryo-embedded brain slices was performed according to standard procedures. The number of occluded and not occluded blood vessels within the ipsilateral hemisphere was counted in every tenth slice for control and 15.0 IU C1-Inh treated mice using a Nikon microscope Eclipse 50i and the % of occluded vessels was calculated.

### Western blot

Cortices or basal ganglia were dissected from the ipsilateral hemisphere of mouse brains and homogenized in RIPA buffer (25 mM Tris pH 7.4, 150 mM NaCl, 1% NP-40) containing 0.1% SDS and 4% proteinase inhibitor (complete protease inhibitor cocktail, Roche). Samples were sonicated for 10 s. Afterwards tissue lysates were centrifuged at 15.0 g for 30 min at 4°C and supernatants were used for bicinchoninic acid (BCA) protein assay and subsequent Western blot analysis. The total lysates were treated with 4x SDS-PAGE loading buffer (final concentration: 62.5 mM Tris pH 6.8, 3% beta-mercaptoethanol, 8% SDS, 15% glycerol) at 95°C for 5 min. 20 μg of total protein was electrophoresed and transferred to a PVDF membrane. After blocking for 30 min with blocking buffer (5% nonfat dry milk, 50 mM Tris-HCl pH 7.5, 0.05% Tween-20) membranes were incubated with the primary antibody at 4°C overnight at the following dilutions: anti-fibrinogen antibody (rabbit, 1:10,000; Acris AP00766PU-N), anti-claudin-5 (mouse, 1:1000; Invitrogen 35–2500), and anti-actin (mouse, 1:500,000; Sigma A5441). After a washing step with TBST (50 mM Tris-HCl pH 7.5, 0.05% Tween-20), membranes were incubated for 1 h with HRP-conjugated donkey anti-rabbit IgG (for fibrinogen) (Dianova) or donkey anti-mouse IgG (for claudin-5 and actin) at a dilution of 1:5000 and were finally developed using ECLplus (GE Healthcare) and quantified by densitometry using the ImageJ software (National Institutes of Health, USA). The relative densities of the protein bands of claudin-5 and fibrinogen were normalized to actin.

### Statistics

All results were expressed as mean ± standard error of mean (SEM). Numbers of animals (*N* = 10) necessary to detect a standardized effect size on lesion volumes ≥20% on day 1 after cortical cryolesion (vehicle-treated control mice vs. mice treated with 15 IU C1-Inh) were determined via a priori sample size calculation with the following assumptions: *α* = 0.05, *β* = 0.2, mean, 20% SEM of the mean (GraphPad Stat Mate 2.0; GraphPad Software). For statistical analysis, the GraphPad Prism 5.0 software package (GraphPad Software) was used. Data were tested for Gaussian distribution with the D’Agostino and Pearson omnibus normality test and then analyzed by one-way analysis of variance (ANOVA) with *post hoc* Bonferroni correction for multivariate analyses. If only two groups were compared, unpaired, two-tailed Student’s *t*-test was applied. *P*-values < 0.05 were considered statistically significant.

## Results

### C1-inhibitor protects from focal brain trauma in a clinically relevant setting

**Figure 1:**
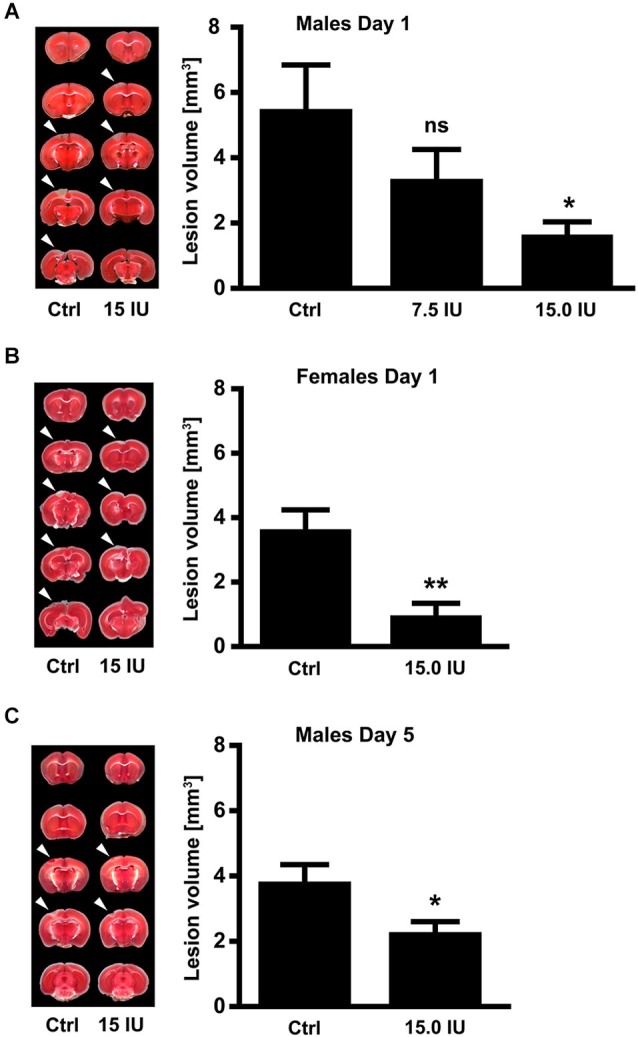
**C1-Inhibitor (C1-Inh) protects against traumatic brain injury in mice of both sexes. (A)** Left panel shows representative 2,3,5-triphenyltetrazolium chloride (TTC) staining of 5 coronal brain sections of 6-week-old male control mice (Ctrl) and 6-week-old male mice treated with 7.5 IU or 15.0 IU C1-Inh. C1-Inh was always applied 1 h after cortical cryolesion and the lesion volume was assessed from TTC staining at day 1. The lesion volume was reduced in a dose-dependent manner with a significant reduction after treatment with 15.0 IU (*n* = 11–13, * *P* < 0.05, One-way analysis of variance with *post hoc* Bonferroni’s Multiple Comparison Test). **(B)** Representative TTC staining and lesion volume of 6-week-old female control and 15.0 IU C1-Inh treated mice, showing a significant reduction in lesion volume after treatment with 15.0 IU at day 1 (*n* = 10–11, ** *P* = 0.0019, Unpaired *t*-test). **(C)** A significant reduction of lesion volume was detectable up to 5 days after treatment with 15.0 IU C1-Inh in 6-week-old male mice (*n* = 10, * *P* < 0.05, Unpaired *t*-test).

To investigate the efficacy of exogenous C1-Inh in acute brain trauma, we chose a cortical cryolesion model in mice. This model induces a rapid breakdown of the blood-brain barrier and is associated with significant edema formation and inflammation (Albert-Weissenberger and Sirén, 2010; Raslan et al., 2012). First, 6-week-old male C57BL/6 mice were subjected to cryolesion and treated with 7.5 IU or 15.0 IU C1-Inh 1 h after trauma (Figure 1). Posttraumatic treatment with 15.0 IU C1-Inh, but not 7.5 IU C1-Inh, significantly reduced lesion volumes by >75% on day 1 as assessed by staining of brain sections with TTC (lesion area: 5.5 ± 1.4 mm^3^ [control] vs. 1.7 ± 0.4 mm^3^ [15.0 IU], respectively; * *P* < 0.05; Figure 1A).

Gender can have a significant impact on the clinical outcome following TBI (Farace and Alves, 2000; Wagner et al., 2005; Ratcliff et al., 2007). Therefore, we also subjected 6-week-old female mice to cortical cryolesion. In line with the results in male mice, treatment of female mice with 15.0 IU C1-Inh 1 h after cryolesion resulted in significantly smaller brain lesions compared with vehicle-treated controls (lesion area 3.6 ± 0.6 mm^3^ [control] vs. 1.0 ± 0.4 mm^3^ [15.0 IU], respectively; ** *P* < 0.01; Figure 1B).

Posttraumatic treatment with 15.0 IU C1-Inh was able to provide sustained protection against TBI. Again, 6-week-old male C57BL/6 mice were subjected to cortical cryolesion and treated with 15.0 IU C1-Inh 1 h after trauma. Assessment of the brain lesion volume after 5 days showed a significant smaller lesion size in the 15.0 IU C1-Inh treated mice compared with vehicle-treated controls (lesion area 3.8 ± 0.5 mm^3^ [control] vs. 2.3 ± 0.3 mm^3^ [15.0 IU], respectively; * *P* < 0.05; Figure 1C).

### Protection from focal brain trauma in C1-inhibitor treated mice results from reduced edema formation, inflammation and thrombosis

**Figure 2:**
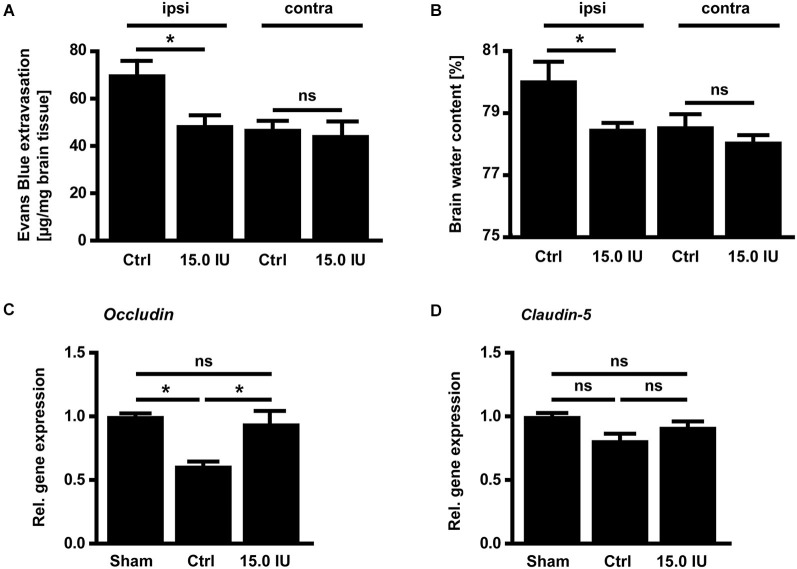
**C1-inhibitor (C1-Inh) treatment results in stabilization of the blood brain barrier. (A)** Vascular leakage on day 1 after cryolesion was significantly decreased after treatment with 15.0 IU of C1-Inh as confirmed by the concentration of Evans Blue detectable in the brain parenchyma (*n* = 6, * *P* < 0.05, ns *P* > 0.05, One-way analysis of variance with *post hoc* Bonferroni’s Multiple Comparison Test, ipsi: ipsilateral hemisphere, contra: contralateral hemisphere). **(B)** Edema formation as reflected by the brain water content in the ipsi- and contralateral hemispheres of control and 15.0 IU treated mice on day 1 after cryolesion (*n* = 6, * *P* < 0.05, ns *P* > 0.05, One-way analysis of variance with *post hoc* Bonferroni’s Multiple Comparison Test). **(C)** Relative gene expression of occludin in the ipsilateral brain parenchyma of control and 15.0 IU treated mice 24 h after cryolesion or sham operation (*n* = 7 or 3 for sham operated, * *P* < 0.05, One-way analysis of variance with *post hoc* Bonferroni’s Multiple Comparison Test). **(D)** Relative gene expression of claudin-5 in the ipsilateral brain parenchyma of control and 15.0 IU treated mice 24 h after cryolesion or sham operation (*n* = 7 or 3 for sham operated, ns *P* > 0.05, One-way analysis of variance with *post hoc* Bonferroni’s Multiple Comparison Test).

Next, we sought to elucidate the underlying mechanisms of this C1-Inh-specific protection in focal brain trauma. C1-Inh plays an important role in the regulation of vascular permeability, probably by inactivating key proteases of the contact-kinin system such as FXIIa or plasma kallikrein (Davis et al., 2010). On day 1 after cryolesion, the integrity of the blood-brain barrier as reflected by the concentration of the vascular tracer Evans Blue leaking into the brain parenchyma was preserved in mice treated with 15.0 IU C1-Inh 1 h after trauma (70.3 ± 5.9 ng/mg [control ipsi] vs. 48.8 ± 4.3 ng/mg [15.0 IU ipsi], * *P* < 0.05; Figure 2A). This finding correlated with significantly less brain edema formation (as assessed by the wet weight-dry weight method) after therapeutic C1-Inh application (80.1 ± 0.6% [control ipsi] vs. 78.5 ± 0.2% [15.0 IU ipsi], * *P* < 0.05; Figure 2B).

In line with a blood-brain barrier stabilizing effect of C1-Inh in TBI, the level of the mRNA encoding for the tight junction protein occludin was downregulated in the brains of vehicle-treated mice compared with sham-operated controls on day 1 after cryolesion (relative gene expression occludin: 1.0 ± 0.02 [sham] vs. 0.6 ± 0.04 [control], * *P* < 0.05; Figure 2C) but occludin mRNA level was preserved in mice receiving 15.0 IU C1-Inh (relative gene expression occludin: 0.9 ± 0.1 [15.0 IU], * *P* < 0.05; Figure 2C). In contrast, no differences in the mRNA levels encoding for another tight junction protein, claudin-5, could be observed between the groups (relative gene expression claudin-5: 1.0 ± 0.03 [sham] vs. 0.8 ± 0.05 [control] vs. 0.9 ± 0.04 [15.0 IU], *P* > 0.05; Figure 2D) indicating selective regulation of specific tight junction proteins by C1-Inh.

**Figure 3:**
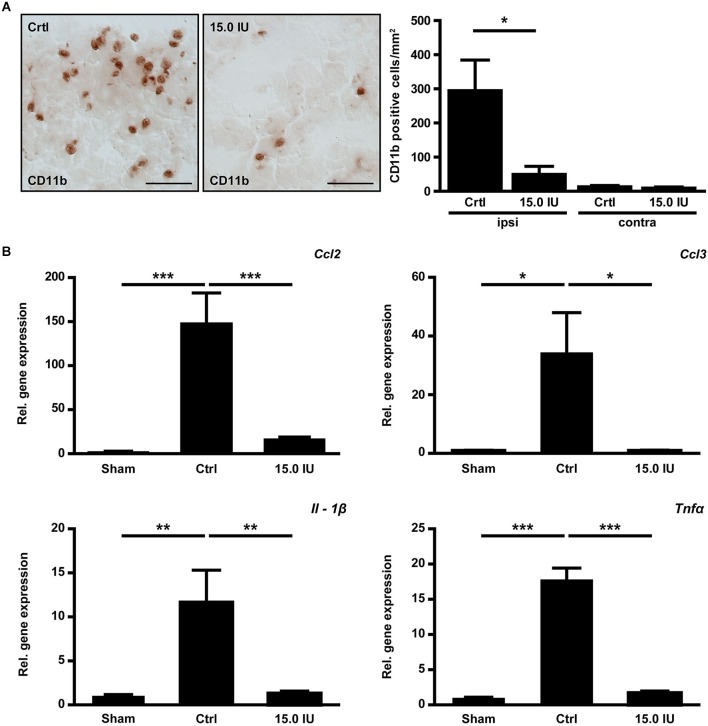
**C1-inhibitor (C1-Inh) treatment results in attenuation of the inflammatory response. (A)** Representative immunohistochemical stainings of cerebral CD11b positive cells on day 1 after cryolesion. Quantification of cell number in control and C1-Inh treated mice revealed less CD11b positive cells per mm^2^ in the lesion site of C1-Inh treated mice (*n* = 5, * *P* < 0.05, One-way analysis of variance with *post hoc* Bonferroni’s Multiple Comparison Test, Scale bar 50 μm). **(B)** Relative gene expression of the genes encoding the proinflammatory cytokines *ccl2*, *ccl3, interleukin* *(IL)-1β*, and *tumor necrosis factor (Tnf)α* in control and C1-Inh treated mice on day 1 after cryolesion. Gene expression of all proinflammatory cytokines were significantly reduced in C1-Inh treated mice (*n* = 5, * *P* < 0.05, ** *P* < 0.01, *** *P* < 0.001, One-way analysis of variance with *post hoc* Bonferroni’s Multiple Comparison Test).

Structural disintegration of the blood-brain barrier facilitates immune cell trafficking and C1-Inh has been shown to inhibit cell migration from the vasculature to sites of inflammation (Cai and Davis, 2003). We therefore quantified the numbers of immune cells invading the injured brain by immunohistochemistry 24 h after the induction of cortical cryolesion. More macrophages/microglia cells had entered the traumatic brains of untreated control mice than of mice that had been treated with 15.0 IU C1-Inh 1 h after TBI (CD11b positive cells/mm^2^ in the lesion site (ipsilateral): 294.6 ± 89.8 [control] vs. 49.4 ± 23.6 [15.0 IU], * *P* < 0.05; Figure 3A). Interestingly, this was paralleled by a significantly reduced mRNA expression of the C-C motif chemokine CCL3 (relative gene expression: 33.9 ± 14.1 [control] vs. 1.00 ± 0.12 [15.0 IU], * *P* < 0.05; Figure 3B). CCL3 is known to promote neutrophil influx especially under inflammatory conditions (Ramos et al., 2005; Johnson et al., 2011; Reichel et al., 2012; de Jager et al., 2013). Accordingly, mRNA expression of CCL2 (monocyte chemoattractant protein 1, MCP-1) was also significantly lower in mice treated with C1-Inh in comparison to vehicle-treated mice (relative gene expression: 147.4 ± 35.1 [control] vs. 15.7 ± 3.3 [15.0 IU], *** *P* < 0.001; Figure 3B).

Next, we analyzed the gene expression profiles of the prototypic proinflammatory cytokines IL-1β and TNFα in the brains of C1-Inh treated mice and controls 24 h after TBI. Both cytokines have been shown to promote traumatic brain damage (Schmidt et al., 2005). Elevation of IL-1β mRNA and TNFα mRNA in the injured hemispheres after cortical cryolesion was less marked in the group receiving 15.0 IU C1-Inh compared with vehicle-treated controls (relative gene expression IL-1β: 11.7 ± 3.4 [control] vs. 1.3 ± 0.1 [15.0 IU], ** *P* < 0.01; Figure 3B; relative gene expression TNFα: 17.8 ± 1.6 [control] vs. 1.7 ± 0.3 [15.0 IU], *** *P* < 0.001; Figure 3B).

**Figure 4:**
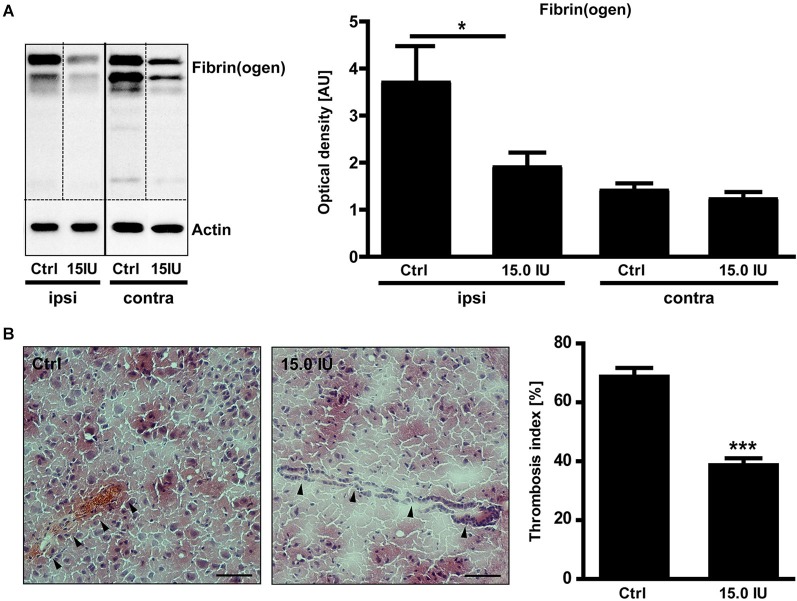
**C1-inhibitor (C1-Inh) treatment results in inhibition of thrombus formation. (A)** Accumulation of fibrin(ogen) in the ipsilateral (ipsi) and contralateral (contra) brain hemispheres of control and 15.0 IU C1-Inh treated mice was analyzed by immunoblotting 24 h after cryolesion, and bands were quantified by densitometry. The representative immunoblot and the quantification shows a significant reduction of fibrin(ogen) in the 15.0 C1-Inh treated mice (*n* = 5–6, * *P* < 0.05, One-way analysis of variance with *post hoc* Bonferroni’s Multiple Comparison Test; AU = arbitrary units). **(B)** Representative H8E staining from traumatic hemispheres of vehicle-treated and 15.0 IU C1-Inh treated mice on day 1 after cryolesion. Occluded vessels (arrowhead in the left panel) were more abundant in control mice when compared to vessels of 15.0 IU C1-Inh treated mice (arrowheads in the right panel). This finding was confirmed by the calculation of the thrombosis index showing a highly significant reduction of occluded vessels in 15.0 IU C1-Inh treated mice on day 1 after cryolesion (*n* = 4, *** *P* < 0.001, Unpaired *t*-test; Scale bar 50 μm).

C1-Inh also blocks FXIIa, the prime activator of the intrinsic pathway of blood coagulation (Davis et al., 2008). Therefore, we additionally analyzed the impact of C1-Inh on the thrombotic activity after cortical cryolesion. The amount of fibrin(ogen) detected by immunoblot in the traumatic hemisphere of C1-Inh treated mice was significantly reduced on day 1 after TBI compared with controls (mean optical density: 3.7 ± 0.8 [control] vs. 1.9 ± 0.3 [15.0 IU], * *P* < 0.05; Figure 4A).

Immunohistochemistry consistently demonstrated intravascular fibrin(ogen) deposits that occluded brain vessels in untreated mice and markedly reduced fibrin(ogen) deposits in mice treated with C1-Inh (Figure 4B). Accordingly, histological sections of lesioned brain tissue from untreated mice showed numerous occlusions of vessel lumina (Figure 4B). In comparison, the microvascular patency was significantly increased in mice receiving C1-Inh (thrombosis index: 69.5 ± 2.2 vs. 39.7 ± 1.7, *** *P* < 0.001).

## Discussion

The salient finding of the present study is that plasma-derived C1-Inh protects from focal brain trauma in different settings relevant to the clinical situation. C1-Inh reduced cortical lesion volumes by nearly 75% in male mice even when applied 1 h after the onset of trauma. Female mice were similarly protected and the beneficial effect was preserved at later stages after trauma. The specific anti-inflammatory and antithrombotic properties of C1-Inh appear to mediate this powerful neuroprotection.

Recent studies indicate that the contact-kinin system is activated after brain trauma under experimental conditions (Albert-Weissenberger et al., 2013). Trabold et al. (2010) found increased levels of bradykinin in the brains of mice subjected to controlled cortical impact and genetic depletion of bradykinin receptor 2, but not bradykinin receptor 1, led to smaller contusion volumes and a better functional outcome 7 days after TBI as compared with wild type mice. In the cryolesion model (Raslan et al., 2010) as well as after diffuse head trauma (weight drop injury) (Albert-Weissenberger et al., 2012), bradykinin receptor 1 seems to dominate over bradykinin receptor 2 but again blocking of bradykinin signaling was neuroprotective in both models. Moreover, treatment with the plasma kallikrein inhibitor aprotinin caused a significant reduction in brain swelling in rabbits which had undergone cold injury (Unterberg et al., 1986). Accordingly, the expression of kininogen was increased in rat brains following fluid percussion injury (Ellis et al., 1989). Auer and Ott (1979) described a rise of proteolytic enzymes in the cerebrospinal fluid of patients with severe head trauma which correlated with overall mortality and which was reversible by aprotinin. However, comprehensive data on the activation status of the contact-kinin system in trauma patients is not available.

C1-Inh is a potent inhibitor of plasma kallikrein, a key enzyme of the contact-kinin system responsible for the release of proinflammatory bradykinin from kininogen (Björkqvist et al., 2013). In line with its antiinflammatory mode of action, C1-Inh stabilized the blood-brain barrier and reduced edema formation after focal cryolesion, an effect that could be ascribed to preserved tight junction protein expression. In addition, mice treated with C1-Inh expressed less IL-1β and TNFα after TBI. IL-1β and TNFα are regarded as a prototypic proinflammatory cytokines known to aggravate traumatic brain damage (Morganti-Kossman et al., 2002; Helmy et al., 2011). Also, significantly fewer macrophages/activated microglia invaded the damaged brains of C1-Inh treated mice in comparison to vehicle-treated controls. Macrophages/microglia are known to be involved in lesion growth following brain injury by producing free radicals and numerous other neurotoxic factors (van Buul and Hordijk, 2004). Several potential mechanisms might account for the anti-migratory effects of C1-Inh in TBI including preservation of blood-brain barrier integrity, binding of cell adhesion molecules (Cai and Davis, 2003), or lowering of chemoattractant factors such as CCL2 and CCL3.

Whereas the anti-inflammatory potential of C1-Inh is well established in a great variety of disease models like sepsis (Begieneman et al., 2012; Heydenreich et al., 2012; Mejia and Davis, 2012), ischemia/reperfusion injury (Horstick et al., 1997; Lehmann et al., 2000; Heydenreich et al., 2012), and spinal cord injury (Tei et al., 2008) the present description of C1-Inh as a powerful antithrombotic compound in TBI is novel and further adds to our understanding of this multifaceted molecule. Of note, the relevance of thrombotic processes in TBI has only recently been recognized. *In vivo* fluorescence microscopy of the brain revealed that microthrombi occluded 70% of venules and 33% of arterioles after controlled cortical impact in mice indicating that the immediate post-traumatic decrease in peri-contusional blood flow is mainly caused by progressive microthrombosis (Schwarzmaier et al., 2010). In addition, intravascular clotting has been described in the same model also at later stages of lesion development, i.e., until day 15 (Lu et al., 2004). Interestingly, platelets can bind to leukocytes and endothelial cells during TBI and this interaction further enhances dysfunction of the neurovascular unit (Schwarzmaier et al., 2010). Similar observations were recently made after experimental cerebral ischemia leading to a redefinition of ischemic stroke as a “thrombo-inflammatory” disease (Nieswandt et al., 2011). The antithrombotic properties of C1-Inh are probably mainly due to its inhibitory action on FXIIa, the origin of the intrinsic coagulation cascade (Davis et al., 2008). However, other mechanisms might contribute as well. For instance, C1-Inh has been shown to directly inhibit thrombin activity on vascular endothelial cells via binding to selectins (Caccia et al., 2011). Moreover, C1-Inh infusions can reduce platelet activity in hereditary angioedema patients and after blood xenotransplantation (Fiane et al., 1999; Coppola et al., 2002).

Longhi et al. (2009) tested the same plasma-derived C1-Inh formulation (Berinert®) at an identical dose (15 IU) in the controlled cortical impact model in mice. In line with our results, C1-Inh significantly reduced lesion size and in addition improved neurological outcome up to 4 weeks after trauma. Here, the neuroprotective effect was greater when C1-Inh was applied already 10 min post injury compared with a delayed application regimen (1 h post injury). Moreover, the impact of C1-Inh on inflammatory processes and thrombus formation was not addressed in this study.

Interesting from a translational perspective, C1-Inh is for many years in clinical use for the treatment of hereditary angioedema, so far without any major safety concerns (Keating, 2009; Banerji, 2010). However, substitution of naturally lacking C1-Inh in individuals with angioedema obviously represents a different situation compared with rising of C1-Inh levels above the normal range in trauma patients. Moreover, measuring of C1-Inh plasma levels in mice revealed that the terminal half-life is between 9.0 and 9.5 h (Dickneite, 1993; Caliezi et al., 2000) while in humans, the mean half-life of C1-Inh was 62 h after intravenous administration and 120 h after subcutaneous administration (Martinez-Saguer et al., 2014). Finally, findings from animal models cannot be easily transferred to the human situation in particular in the case of cortical cryolesion which only mimics certain aspects of brain trauma such as excessive edema formation and inflammation (Albert-Weissenberger and Sirén, 2010). Nevertheless, the fact that C1-Inh mediates neuroprotection in a broad array of neurological disease models is reassuring (Begieneman et al., 2012; Heydenreich et al., 2012; Mejia and Davis, 2012) and underpins its potential applicability in the clinic.

In summary, C1-Inh ameliorates trauma-induced neurodegeneration in different clinically relevant scenarios by counteracting “thrombo-inflammation”. Therefore, C1-Inh might become an attractive candidate to combat TBI and other neurological conditions associated with inflammation and thrombosis.

## Conflict of interest statement

The authors declare that the research was conducted in the absence of any commercial or financial relationships that could be construed as a potential conflict of interest.
